# Analysing implementer narratives on addressing health inequity through convergent action on the social determinants of health in India

**DOI:** 10.1186/s12939-015-0267-7

**Published:** 2015-11-17

**Authors:** Devaki Nambiar, Arundati Muralidharan, Samir Garg, Nayreen Daruwalla, Prathibha Ganesan

**Affiliations:** Public Health Foundation of India, Plot No. 47, Sector 44, Institutional Area, Gurgaon, National Capital Region 122002 India; Chhattisgarh State Health Resource Centre, Raipur, Chhattisgarh India; The Prevention of Violence Against Women and Children Programme, Society for Nutrition, Education, and Health Action (SNEHA), Mumbai, India

**Keywords:** Social Determinants of Health, India, Health inequity, Implementation

## Abstract

**Background:**

Understanding health inequity in India is a challenge, given the complexity that characterise the lives of its residents. Interpreting constructive action to address health inequity in the country is rare, though much exhorted by the global research community. We critically analysed operational understandings of inequity embedded in convergent actions to address health-related inequalities by stakeholders in varying contexts within the country.

**Methods:**

Two implementer groups were purposively chosen to reflect on their experiences addressing inequalities in health (and its determinants) in the public sector working in rural areas and in the private non-profit sector working in urban areas. A representing co-author from each group developed narratives around how they operationally defined, monitored, and addressed health inequality in their work. These narratives were content analysed by two other co-authors to draw out common and disparate themes characterising each action context, operational definitions, shifts and changes in strategies and definitions, and outcomes (both intended and unintended). Findings were reviewed by all authors to develop case studies.

**Results:**

We theorised that action to address health inequality converges around a unifying theme or pivot, and developed a heuristic that describes the features of this convergence. In one case, the convergence was a single decision-making platform for deliberation around myriad village development issues, while in the other, convergence brought together communities, legal, police, and health system action around one salient health issue. One case emphasized demand generation, the other was focussed on improving quality and supply of services. In both cases, the operationalization of equity broke beyond a biomedical or clinical focus. Dearth of data meant that implementers exercised various strategies to gather it, and to develop interventions – always around a core issue or population.

**Conclusions:**

This exercise demonstrated the possibility of constructive engagement between implementers and researchers to understand and theorize action on health equity and the social determinants of health. This heuristic developed may be of use not just for further research, but also for on-going appraisal and design of policy and praxis, both sensitive to and reflective of Indian concerns and understandings.

## Background

Equality has been defined as a fundamental value in the 2000 Millennium Declaration, and yet, the past decade has seen an intensification of inequalities within and across countries [1]. In 2005, the WHO Task Force on Research Priorities for Equity in Health and the WHO Equity team noted that much of the existing research lacked policy-relevant synthesis, calling for research into global factors and processes affecting health equity, the effects of societal and political structures in relation to health, relationships between individual circumstances and contextual factors, as well as health system factors and policy interventions that address equity [2].^1^

In the seven years that followed, a great deal more research on inequity and inequality has emerged, also finding explicit articulation in the United Nations Sustainable Development Goals. These efforts have been summarized in the Task Force’s follow up review [3]. Here, they made the following recommendations: “focus on identifying and evaluating policy options, propelled by the search for what works in practice to reduce health inequities; empower research managers, policy makers, and funders to generate national and regional research agendas and fund priorities that address equity and health; and support the strengthening of collaborations, capacities, and methods to do so” [3]. This paper seeks to understand what works in practice to reduce health inequities. It focuses on research and policy options, embedding our search in ongoing praxis in a manner that enables interactions between researchers, policymakers and funders (similar efforts have been undertaken for instance, in South Africa) [4]. As a process, writing the paper sought to empower those engaged in research to build capacity in understanding field level realities and those in praxis to build capacity in analysis methods.

## Framing the issue: inequity and the Social Determinants of Health (SDH)

The Report of the World Health Organisation’s Commission on the Social Determinants of Health declared that social gradients in health outcomes are due to the unequal and structural distribution of power [5]. This has been validated by the UNDP’s Synthesis Report on Inequalities released in 2013, which argues that inequalities are shaped by structural barriers in economic, social, environmental and political domains, and are also mutually reinforcing [6]. Prior research and theorising has also shown that inequalities persist due to historical disadvantage reinforced by social, political and economic factors [7]. In a recent review, it has been pointed out that “strengthening the determinants of health and well-being beyond the provision of health care services, such as housing, social support, income and food security, is essential to prevent or reduce inequities in health” [8]. Indeed, a great deal of global research and advocacy on health inequalities has been prompted by Knowledge Networks of the Commission on Social Determinants of Health over the past decade.

In India, health reform efforts also take cognizance of the importance of social determinants in addressing inequality. The vision propounded by India’s High Level Expert Group on Universal Health Coverage (UHC) in 2011 was broad, opening itself up to include the Social Determinants of Health (SDH) [9, 10]. For coverage to be universal, it has to cover a number of intersecting identities and circumstances that reflect SDH, such as place of residence (rural/urban), race, ethnicity, religion, caste, occupation, and gender, among others.^2^ In its SDH chapter, the HLEG has proposed the development of a “Health Equity Surveillance Framework” or Health Equity Watch, to “map the nation’s progress in closing gaps in health equity” [9]. This is itself an exercise in transparency, allowing us not only to keep a watch on health reform, but also be reflexive about our engagement in and contributions to it.

There is a large academic canon looking at the issue of inequality in health and its social determinants, mostly drawn from routinely collected data, such as the National Family Health Surveys and some primary data collection [11–19]. Recently, research consortia have also sought to examine, synthesize, and theorize around Indian research on health equity [20, 21]. In the domain of praxis and policy, however, rather than exploring various determinants, emphasis has been placed on the notion of convergence, defined as “coordinated policy decisions and programme actions in multiple sectors [fields that have proximal or distal effects…]…to achieve a common goal” [22]. Convergence also seeks to “establish a synergy between the government, Non-Governmental Organisations (NGOs), the private sector and the beneficiaries for a progressive realization of the rights of India’s poorest citizens” [23]. As Sharma points out, various forms of convergence have been attempted, including convergence as sharing of human or financial resources from different sectors in a government programme, or collaboration and complementarity between NGOs and the government working in a particular geographic area (for instance, to serve remote tribal populations) [23]. The unification of determinants for joint action, rather than their multiplicity, is how SDH seem more commonly to be acted upon - at least at the policy level- in India [23]. While further examination of the notion of convergence is required, it appears to us to be a frame that emphasizes action and process, rather than concepts and determinants, and is thus of interest.

Debate on health and its social determinants is lacking in India [24], something that Baru and Sivaramakrishnan also observed at the release of the report of the Commission on Social Determinants of Health [25]. They urged informed debate “both within and outside the public health community” on health and its social determinants [25]. In recent work on people-centredness in health policy and systems research (HPSR), authors highlighted that researchers are the key to aggregating, synthesising and analysing available knowledge, but that this role requires close engagement with various actors within the system [26]. A recent paper discussing policy intervention in SDH globally exhorts greater attention towards “understanding the ways in which policy-makers learn from themselves…. Conceptual models are useful techniques in such learning” [27]. Our work proposes to contribute to greater and more meaningful, mutually constitutive and constructive interaction across researchers, policymakers and practitioners in public health, seeing it as a critical next step in addressing health inequity and the social determinants of health. More narrowly, the objective of this work was to heed the Task Force’s call to understand “what works in practice to address health inequity.” We undertook this through critical and collaborative analysis of narratives from two successful attempts at addressing SDH in India to conceptualise what their shared features are and what lessons they offer.

## Methods

In June of 2013, a national consultative workshop was convened by the World Health Organisation’s Country Office in India and the Public Health Foundation of India on developing an Indian ‘Health Equity Watch.’ It was observed that considerable effort and attention has been directed towards this issue in praxis [28]. The workshop comprised presentations and participation of stakeholders from three countries and eight Indian states, including government representatives, technical experts, and social change agents working at the grassroots. At the meeting, it became increasingly clear that there was a disjuncture between policy and praxis efforts and research work in this area; we needed to more closely interact to heed the global call for policy-relevant action to address health inequity [6]. We therefore sought a methodology that served the purpose of bringing stakeholders together and of shedding light on our topic of interest. We arrived at case study methodology, defined as “the study of the particularity and complexity of a single case, coming to understand its activity within important circumstances” [29].

The National Collaborating Centre on Determinants of Health, Canada seeks to bring together research and praxis in relation to health equity and intersectoral action, and as such overlaps greatly with our goals and interests. They define case study method as a form of “problem- based learning… [which] involves all the participants in actively defining the problem and developing a range of solutions” [30]. The June 2013 workshop led a larger group of stakeholders interested in SDH and health equity towards the idea of convergence - the possibility that cases of convergent action were underway and had to be more closely understood. But convergence of what? For what? In order to answer this, case study method was explicitly chosen to study existing convergence efforts because of its utility as a learning tool, its emphasis on real-life situations and problem-solving, and the possibility of adapting and expanding lessons in other contexts.

The methodology for case studies we followed, as aforementioned, was specific to our goals and context. Thus, in place of the usual standards for the reporting of qualitative research for case studies, we indicate and justify the actual process we followed. As recommended by the NCCDH methodology, at the June 2013 meeting, various implementer groups were purposively chosen to reflect on their experiences acting to address inequalities in health (and its determinants) in the public sector and in the private non-profit sector. Unlike the NCCDH process, however, we used our workshop as a stepping-stone to build a guideline for the development of narratives that would then become case studies developed collaboratively with implementer groups. Our goal as researchers was not just to document experiences, but to engage practitioners as co-authors/co-producers of the knowledge/reflection, following from the principles of Participatory Action Research (PAR) [31] and qualitative comparative analysis [32].

PAR is defined as having the following key features: “Firstly, it transforms the role of those usually participating as the subjects of research and involves them instead as active researchers and agents of change. Participatory action research aims to overcome the separation between subject and object. Those affected by the problem are the primary source of information and the primary actors in generating, validating and using the knowledge for action…. Secondly, it involves developing, implementing, and reflecting on actions as part of the research and knowledge generation process” [31]. Having involved two such groups, we undertook qualitative comparative analysis, which summarises qualitative evidence from individual studies along common categories (what we called ‘provocations’^3^) and compares them [31, 32]. Applying principles of PAR, therefore we were “moving from describing to search for causes, with direct reflection on problems by those affected and testing the understanding built to learn from action” [31].

The first implementer group was the Chhattisgarh State Health Resource Centre, a technical support agency which created monitoring registers maintained by Village Health and Sanitation Committees that contained routine information on education, sanitation, and health outcomes. The second was the Society for Nutrition Education and Health Action, a non-governmental organisation operating in Mumbai slums, which has converged advocacy and design of appropriate interventions related to violence against women from slum communities, in concert with their communities, health care providers, law enforcement, and the judiciary. Based on prior action-research on health inequalities, and a review of the national and international literature, two lead authors developed provocations (questions or statements listed out to incite critical thinking and writing), pertaining to the genesis of the programme (including influences, beneficiary groups, duration, location, scale, and stakeholdership), knowledge gaps and needs, actions taken (including influencing events, opportunities and threats), as well as what implementers saw as key lessons, challenges and future steps with relation to their cases.

Each implementer group developed a narrative in response to these provocations, detailing how they operationally defined, monitored, and addressed health inequity in their work. A narrative in our work was therefore defined as a written retelling of events and activities with which implementers were involved that corresponded with the provocations, focusing on as detailed description as could be given. These narratives were content analysed by the two lead authors to draw out contextual factors, focus of intervention, drivers of health inequity in each case, rationale for intervention, strategies used to address inequity, the involvement of state and non-state actors, as well as challenges faced in each case. Analysis resulted in the creation of a heuristic - a visual depiction to assist our conceptual understanding - to describe convergence in addressing health inequalities (see Fig. 2).

Throughout our process, we have aimed to establish rigour in keeping with the demands of PAR [31] and qualitative research principles [33, 34]. The provocations presented here, as well as the detailing of process is meant to serve as an audit trail to ensure reliability and confirmability across other studies and situations. We also sought as much detail as possible so that analytic generalisations could be identified across our two cases [35], reflective of both variations and similarities in findings. The narrative text is presented here in detail so as to demonstrate how analyses were arrived at and allow our method to be transferable to other cases. The main product of analysis, our heuristic, was subject to validation via member-checking [36], i.e. analysis was shared with narrative authors and one other individual involved with implementing the programme to ensure emerging analyses were reflecting actual understandings and experiences. This was a means of establishing credibility of analysis and findings. Finally, we have been reflexive about the role of all participants in this work and in the analysis, so as to adequately apply PAR methods: ownership and authorship of this work is thus shared with implementer groups (they are third and fourth authors of this manuscript).

## Results

We present narratives developed by co-authors from implementer groups. Our analysis of these narratives follows in the discussion section.

### Case narrative 1: Community watch and action on health and its social determinants: evolution of the Swasth Panchayat Programme in Chhattisgarh

The Government of Chhattisgarh’s Swasth Panchayat Yojana was built upon the base prepared by the Mitanin Programme, a Community Health Worker (CHW) programme initiated by Government of Chhattisgarh in 2002. It has nearly 67,000 CHWs called Mitanins covering almost all the rural habitations of the state.^4^ The programme is recognized as a highly successful CHW programme and is often credited with the unprecedented decline achieved by the state in its rural Infant Mortality Rate [37].

The role defined for the CHWs in the Mitanin Programme set the state on the path leading to the emergence of the Swasth Panchayat or the Healthy Panchayat scheme. The concept of health as taught to Mitanins in their curriculum included emphasis on SDH. The role of Mitanin therefore extended beyond health education and curative elements to include mobilisation of women as well as locally elected bodies, and facilitating systematic local health planning and action [38]. Experiments in tribal-dominated Koriya district of the state had shown the efficacy of Mitanins in organising community watch on nutrition programmes through neighbourhood committees [39].

In 2006, the Swasth Panchayat scheme was launched with the objective of enabling local communities and Panchayats (village councils) to assess the situation of their health, identify gaps, plan and execute actions to address the gaps. Mitanins and members of Panchayats across the state were trained jointly on critical aspects of community health and how to collect data on them [40]. Involvement of local community in data collection was aimed at creating an opportunity for them to assess their health situation. Disaggregated data was collected for each habitation on 26 indicators. It was recorded in a Panchayat score card. The data set was computerized to compute Panchayat level composite scores. Awards were given to the top ranking Panchayats in each block. The scheme covered all 146 rural blocks of the state and Swasth Panchayat data collection actually took place in nearly 80 % of the 70,000 rural habitations in the state. Four rounds were carried out between 2006 and 2011.

Based on these data, comparisons were possible across habitations on objective health indicators. Stark inequalities were identified between habitations in terms of their access to health, nutrition, drinking water and education services. Habitations with poorer communities often had lower access to services. Inter-Panchayat comparisons were also very instructive. Panchayats located in more remote locations and having predominantly tribal population usually got very low scores. The scores therefore did not realistically reflect a Panchayat’s performance in acting on health issues. The score was much more a reflection of the extent of inequity faced by a Panchayat in comparison to others. Notwithstanding this, a challenge emerged for Swasth Panchayat scheme around how to enable local communities to understand and make use of the information. It required a community level platform which focused on health and encouraged participation of Panchayats in it.

In 2007–08, the state constituted Village Health Sanitation and Nutrition Committees (VHSNCs), a key intervention promoted under the National Rural Health Mission (NRHM). The VHSNC provided the ideal platform for promoting a ‘Community Watch’ on health and social determinants. In 2008, NRHM had also introduced another component called ‘Community Based Monitoring’ which brought in civil society groups with the primary objective of organising a community watch over health services.

From 2009 onwards, Chhattisgarh decided to integrate all these interventions under the Swasth Panchayat scheme with the state bearing around two-thirds of the cost. For two years, VHSNCs tried to utilize the information available through Swasth Panchayat indicators for identifying local health gaps and planning collective action to address them. Based on this experience, the indicators used in Swasth Panchayat survey were simplified so that rural communities could understand them easily. In 2011, the annual Swasth Panchayat survey was transformed into a Village Monitoring Register that was updated monthly in VHSNC meetings. It allowed communities to have more continuous monitoring on key issues.

Village Monitoring Registers monitored health status, service access and determinants using basic counts in the last month of the following:

1. Health status (comprising mortality - infant mortality, maternal mortality, and by common causes like malaria, diarrhea, TB, pneumonia etc.; morbidity - due to common causes; malnutrition; and violence against women);
2. Access to local health services (including immunisation, free drug provision, referral transport, and use of bed nets); and
3. Access to underlying determinants of health including food, water, sanitation and education, again linked to government schemes and entitlements (including functionality of hand-pumps, toilets, girls’ school attendance, mid-day meals, rural employment guarantee wage payment, provisions of food under the Integrated Child Development Scheme).

This data is being used at state level to assess health inequity [41]. Particularly the mortality register has been used to triangulate government data of all-cause and specific-cause mortality. This creates an alternative source of evidence of inequity especially where Governments often grossly under-report cause-specific mortality (e.g. for malaria or diarrhoea). Further, this data has also been combined with community feedback data gathered on expenditure and facility level care.

Moreover, this monitoring experience is an example of how data on health equity could be used; the Village Monitoring register data was connected to Village Health Action plans in a step-wise process of identifying a gap, its cause, a response, responsibilities of different stakeholders, and timeline for joint action. Internal assessments have shown that by 2012, around two-third of the villages in the state had started using this methodology actively [41].

### Case narrative 2: Multi-level and multi-sectoral action to address gender based violence in Mumbai’s urban slums: the prevention of violence against women and children program, SNEHA

The Society for Nutrition Education and Health Action (SNEHA) was founded in Mumbai in 2000 by the former Dean of Sion hospital. The organisation was established in the Urban Health Center of Sion hospital, serving Dharavi, a cluster of 90 slum settlements, and other non-slum communities in the area. In its early days, a solitary social worker operated out of a single room. Soon after, a crisis centre for women and children in distress was established in 2000 to provide a safe space and a supportive environment for those experiencing violence. SNEHA also convinced the Municipal Corporation of Mumbai to allot some hospital beds for abused women in need of shelter. SNEHA offered psychotherapeutic and psycho-social interventions to women in the wards as well as those who came to the centre for help.

In 2001–2002, SNEHA conducted a survey in Dharavi, finding that almost three in four women respondents living in these slums reported experiencing some form of violence. A fraction of these women sought assistance, medical or psycho-social, at the hospital. Struck by this, SNEHA decided to extend its work beyond the crisis centre in the hospital and reach women directly in the community.

The organization was mindful, from an early stage, that violence interventions must be two-fold: medical treatment and psycho-social support for victims complemented by preventive and promotive activities in slum communities. The awareness generation activities informed women that medical, legal, and social support services were available for those in violent situations. They also focused on family and community, sowing the seeds for making violence a public concern. SNEHA’s earlier work had exposed its staff to the uninformed response of the health system towards abused women presenting with injuries at health facilities. Law enforcement agencies also had to be oriented; they were perceived to be insensitive towards women reporting violence and therefore distrusted by women, and seen to be fuelling unequal gender norms that drove women back to an abusive environment.

From 2004, SNEHA started work with health care providers, specifically doctors and nurses in tertiary care facilities to sensitize them on the dimensions of gender-based violence, and worked with them to develop referral mechanisms for abused women and children. By 2010, SNEHA had established two more crisis interventions centres in tertiary level public health facilities. As more women sought assistance at the crisis centres, SNEHA found itself ill-equipped to provide legal assistance. The organization therefore hired and trained lawyers to provide women with legal aid. Working with law enforcement agencies remained one of SNEHA’s biggest challenges. India’s Protection of Women against Domestic Violence Act, 2005 (PWDVA) provided a platform for more active engagement with the police as SNEHA was deemed a service provider under this Act. The organization used this opportunity to further its work with the police and in 2012 launched an intervention that sensitizes and trains police to respond to violence in a timely, well-informed and sensitive manner. Simultaneously, SNEHA has intensified community outreach activities forming women’s and youth (adolescent girls and boys) groups that conduct door to door, housing lane-by-lane, and community-level activities to increase knowledge about physical, sexual, emotional, and financial forms of violence, and actively challenge unequal gender norms that underlie violence against women.

Violence is often perceived as a private or family matter, yet SNEHA’s programme for the Prevention of Violence against Women and Children (PVWC) has worked to make violence a public concern, highlighting the responsibility of various stakeholders to prevent and respond to violence in a timely, sensitive, and comprehensive manner. Today, SNEHA’s program on violence, includes individual psycho-social, couple and family counselling services by trained counsellors and social workers, legal aid services, and medical treatment and police intervention for abused women and children. SNEHA’s work reaches over 900,000 people living in slum and non-slum areas across Mumbai.

SNEHA believes that its work is limited by the paucity of data on violence. The National Family and Health Surveys (NFHS) provide some data on the prevalence of domestic violence, help-seeking behaviour, and attitudes towards violence against women, yet the focus is more on married women, overlooking the needs of unmarried women, youth, and children [42]. Smaller community-based and facility-based studies do provide some insights, but again are limited in scope. Health facility records, police records, and legal records often do not provide reliable estimates on the incidence and information on the nature of violence faced by women.

With respect to using knowledge, there are two key interrelated issues: first, violence is hard to measure, given its highly sensitive, personal, and varied nature. Violence within the realm of marriage is considered to be a private, family matter, and is often justified. Second, and on a related note, the proportion of women reporting violence may be underestimated given poor help-seeking behaviours. The Third National Family Health Survey (NFHS 3) for Maharashtra found that among those experiencing physical violence, only 0.4 % of sought medical aid and 4.6 % sought police assistance [42]. SNEHA found little qualitative exploration of the perceptions of and the barriers faced by health care providers, police, and the legal systems that limit or enhance their ability to provide timely, appropriate, and responsive assistance in instances of violence [43], especially for women living with disabilities [44]. Such insights are needed, and can help tailor interventions to address these barriers.

SNEHA’s multi-sectoral and multi-level approach to addressing violence has created a platform for making violence a serious public concern at various forums. A major challenge that now lies before SNEHA is facilitating referrals between these different stakeholders for a coordinated and sustainable response to violence. Another challenge is developing appropriate methodologies to assess the impact of these various interventions on preventing and/or addressing violence in communities.

## Discussion

**Fig. 1:**
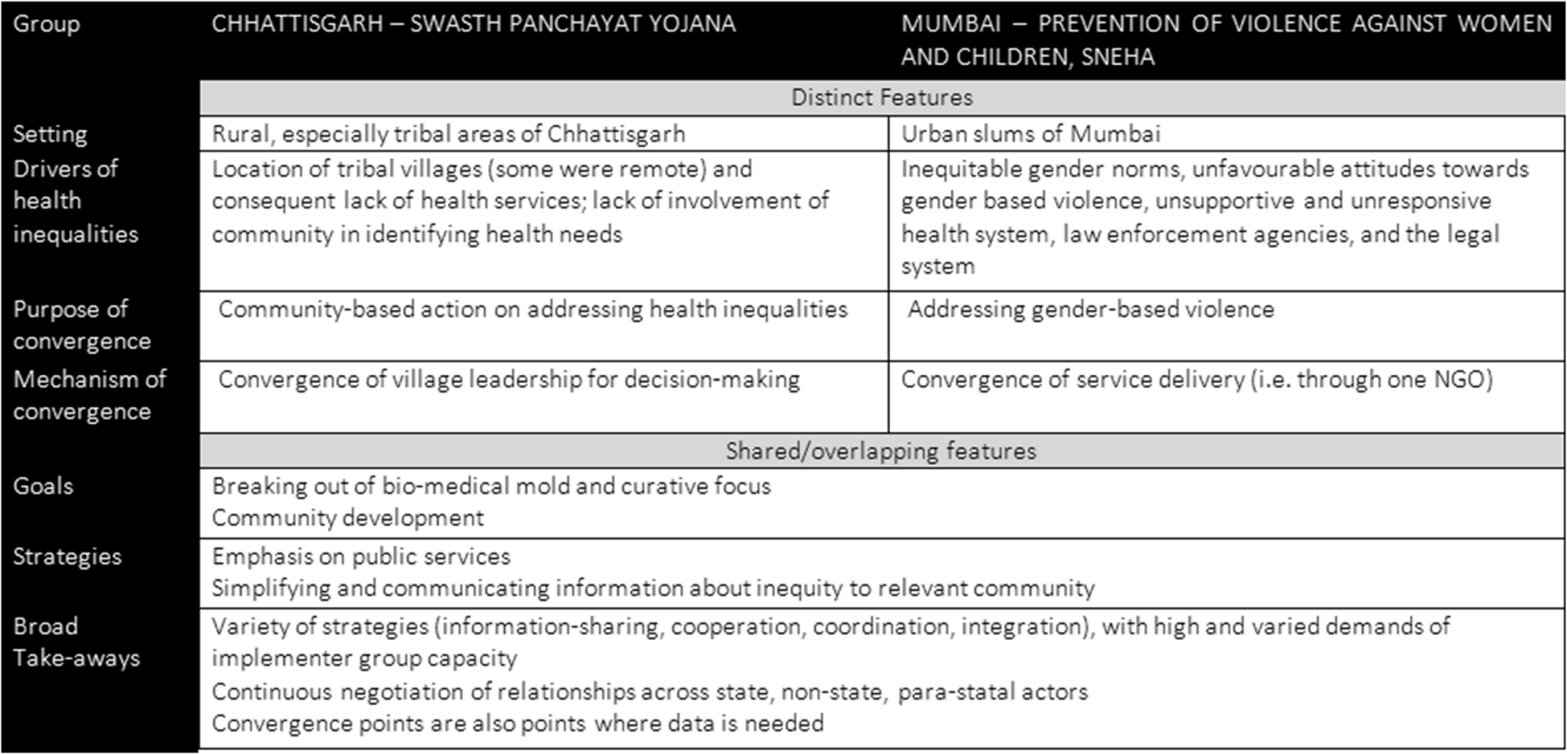
Distinct and shared/Overlapping features of case studies

**Fig. 2:**
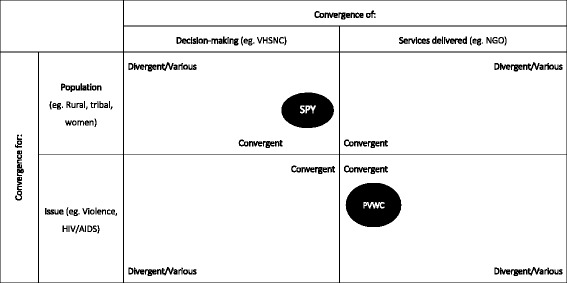
Mechanisms for convergence in addressing health inequities. Note: SP = Swasth Panchayat Yojana of the State Health Resource Centre, Chhattisgarh; PVWC: Prevention of Violence Against Women and Children Programme of the Society for Nutrition Education and Health Action (SNEHA). Source: Authors

Key features distinguish the two case narratives presented that may in fact point towards the articulation of a framework (see Figs. 1 and 2). The application of the Swasth Panchayat Yojana was centred on rural and tribal areas across an entire state whereas the work of SNEHA’s violence prevention activities was concentrated in an urban slum pocket in the city of Mumbai. There were, therefore, major variations in geography, scale, as well as the provenance of implementer group: the Swasth Panchayat scheme is a government initiative while SNEHA is non-governmental.

What brings the two cases together despite all these differences is the theme of convergence - by way of both purpose (labelled “convergence for” in Fig. 2) and mechanism (labelled “convergence of” in Fig. 2). The first case entailed providing support and tools to communities to track and act on health inequalities while the latter entailed the expansion of the types of support offered to survivors of violence and those vulnerable to it. The Swasth Panchayat Yojana involved convergence of decision-makers (i.e. the Village leadership) for a population (i.e. the village), SNEHA’s initiative converged service delivery (i.e. through the NGO) on a particular issue (i.e. gender-based violence). While in Chhattisgarh, the focus was on increasing effective demand for service improvement, in Mumbai, efforts were simultaneously geared towards enhancing quality and supply of services based on expressed and assessed needs of users. In the case of Chhattisgarh, implementation at scale was made possible by the foundation of the community health worker programme and the bringing together of various government schemes at the community level. In Mumbai, to the extent that efforts were initiated by an NGO, much of the expansion occurred within the organisation itself to support the various needs of beneficiaries, gradually making possible an expansion of coverage to a larger number of people over time.

In terms of similarities, in both cases, groups quite early on were clear that understanding health inequalities requires breaking out of a biomedical, clinical, or curative focus. Community development was a major theme that cut across both the case narratives precisely because any action on SDH requires close partnership and ownership of communities. For example, while the village plays a vigilance and oversight role in Chhattisgarh, the emergence of gender-based violence as an issue itself emerged from the community in the case of SNEHA. In terms of strategies, in both cases, engagement with public services was a central focus. Further, implementers had to confront a lack of systematic data collection and relied, instead, on the provisions of policies and schemes to gather information, data, and develop action strategies. Both initiatives involved a process of simplifying and communicating information on inequity to relevant groups (village-dwellers in Chhattisgarh, police officers and health care providers in Mumbai slums).

We also noted that the implementer groups had to exercise a variety of strategies, incrementally and cyclically, to be able to make use of data, fill gaps where data did not exist, and develop strategies based on both data conclusions and gaps. The demands on the skillsets and capacities of implementer groups, therefore, were extremely high. Each group also had to negotiate a series of relationships across state, para-statal, and non-state actors and their respective programmes, in order to operate.

Shankardass and colleagues’ review of intersectoral action for health describes four patterns of relationships between health and non-health sectors: **information-sharing**, seen as the on-way relaying of information from one sector to others; **cooperation**, where sectors lose some autonomy in the interest of optimally utilising resources together, **coordination**, where policies and programmes within each sector are horizontally adjusted and networked, and **integration**, where new policies and programmes are defined and developed in conjunction with other sectors [8]. What we’re seeing in both cases is a combination of these relationships. For example, while SNEHA engages in extensive information-sharing, their aim in doing this is also for various sectors to adjust programmes of health facilities, law enforcement, and legal action in order to more appropriately prevent and respond to gender-based violence in low-income communities. In their experience, building coordination mechanisms between sectors is an important but challenging task, as few models exist. In the case of SHRC, while the Panchayat Fellow programme may not have been actively developed with direct deliberation with the Panchayati Raj ministry, resources are divested to Panchayats for the purposes of spending on health and sanitation.

Conceiving of action to address inequity as a form of convergence also provides indication on where data availability and data collection points may be organised. For instance, for convergent decision-making structures, data across sectors must be made available to decision-makers. Further, if convergence is around a particular health issue, that is the starting point to map out additional equity stratifiers, data needs, sources, gaps, etc. (a methodology for this has been developed by the World Health Organisation) [45]. In effect, points of convergence, rather than just being a mantra for policy-makers, may offer points of orientation for research design, data collection, and knowledge translation, using a methodology that not just relates to inequity, but also addresses it in practice.

Future research should explore to what extent the forms of convergence observed here are inherently linked to the scale/context in which they were observed: for example, are there forms of population-based convergence on a single decision-making platform in urban areas? Some research point to convergence for instance, to assure a number of entitlements for certain groups of urban informal workers [46], although research has also shown a multiplicity of jurisdictions [47] and relative inattention to the constitutional mandate of local decision-making in Indian cities and towns [48]. While our findings suggest that village-level committees represent a decision-making platform for rural India that can promote convergence, research suggests that there are barriers related to capacity and programme design hindering the use of such ‘decision spaces’ for convergence in other Indian states [49, 50]. This would have to be explored more systematically in future research, particularly in relation to certain issues like land and forest rights.

As regards programming and policy design, the case for convergence is strong, but what is lacking is operational direction on how this may move forward. Here, approaches suggested in the x axis of Fig. 2 may be salient. For instance, we may act to address the many interlinked challenges befalling India’s indigenous populations, detailed at length by a recent High Level Committee [51] appointed by the Indian Ministry of Tribal Affairs, by converging services across the Ministry of Health and Family Welfare, Ministry of Labour and Employment, Ministry of Human Resource Development, and of Women and Child Development. A next step could be to look at issue-population combinations as a starting point (eg. malnutrition among tribal women) and examine what is already being done by various stakeholders and how each Ministry may add to or enhance its contribution. Further, looking at urban water and sanitation could be another example, giving specific definition and policy direction to the recently launched Swacch Bharat Mission, a ‘Clean India’ initiative propounded by the current government. Thus far, the focus on infrastructure in rural areas has dominated sanitation policy and programming. The lack of safe water and adequate sanitation in urban areas affects communities, schools, health care facilities, and worksites. Action is needed across sectors to address infrastructure needs, socio-cultural norms, and individual preferences and behaviours that shape the provision and uptake of these services in various urban settings.

## Conclusion

In conclusion, this exercise has demonstrated on the one hand the possibility of constructive engagement between implementers and researchers to understand - even theorize- action to address health inequity and the social determinants of health. On the other, the heuristic that has emerged may be of use not just for further research, but also for ongoing appraisal and design of policy and praxis, both sensitive to and reflective of Indian concerns and understandings.

## Footnotes

1 We concur with the often-stated distinction between health inequity and inequality [45, 52]. While inequality refers to observable differences between subgroups in a population, inequity is a normative concept, referring to unjust and avoidable differences between persons of different social groups, linked to forms of disadvantage, discrimination and lack of access. While we agree that it may be difficult to measure health inequity directly, action to address health inequity can be deliberately carried out. Addressing health inequity was the focus of this work.

2 The acronym PROGRESS (**P**lace of residence (rural/urban, etc.); **R**ace/ethnicity; **O**ccupation; **G**ender; **R**eligion; **E**ducation; **S**ocioeconomic status; **S**ocial capital/resources) has been proposed as an illustrative or starting point of indicated social determinant stratifiers that should be considered in any analysis of health equity [45, 53].

3 The use of the word “provocations” is deliberate - it comes from the book for implementers entitled *Provocations for Development* by Robert Chambers [54], a major proponent of participatory methodologies. A provocation is meant to provoke, that is to incite, stimulate in a manner that may be heretical and seeks to see things differently.

4 The word “Mitanin” means “friend” or “comrade” in one of the local languages spoken in the state.
